# Remarks upon Mr. M'Donald's Objections and Statement Respecting the Convulsive Epidemic in Cornwall

**Published:** 1814-07

**Authors:** James Cornish

**Affiliations:** Member of the Royal College of Surgeons. Falmouth


*For the Medical and Physical Journal.*

### *Remarks upon Mr. M'Donald's Objections and Statement respecting the Convulsive Epidemic in Cornwall; by* Mr. Cornish.

IN the Medical and Physical Journal for this month, I have just read a paper entitled "Observations on Mr. Cornish's Account of a Convulsive Epidemic in Cornwall, by Mr. James M'Donald." As Mr. M'D. has endeavoured to fix on me a charge, which no man who is confident in his veracity can overlook, I trust you will do me the justice to enable me to vindicate myself from so foul an imputation. The charge to to which I allude is conveyed in the last paragraph of Mr. M'D.'s paper, where he says, "Without supposing that Mr. C. has *intentionally* misrepresented any of the facts recorded in his paper, it is proper to observe that three accounts which now lie before me, written by eye-witnesses, who are men of sound sense and strict integrity, *differ materially* from that which he has given." Had Mr. M'Donald candidly stated in what particular circumstance I had been guilty of misrepresentation, I should have been able to reply to it directly; but, as he has sheltered himself under a general charge, I am at a loss to discover in what respect my account "differs materially." If the difference between my account and that which Mr. M'D. has received from "eye-witnesses of sound sense and strict integrity" be *material*, it must be in some of the following circumstances. No such disease, or no such symptoms, have existed as I have described; or the disease has not prevailed to the extent asserted; or no different opinions have been entertained respecting its cause; or the exhortations, &c. to which the affected individuals had been exposed were not of the nature I have stated. These appear to me to be the only circumstances wherein a difference considerable enough to amount to a "misrepresentation" can have arisen. That the symptoms were as I have described, that different opinions have prevailed respecting their cause, and that the exhortations were of the nature I have stated, are facts, for which I am personally responsible, and of which, if necessary, I can adduce ample proofs. That the disease has prevailed in the places, and to the extent mentioned, I have the authority of the methodists themselves for asserting, which authority I suppose Mr. M'D. will not hesitate to receive.

The manner in which Mr. M'D. proceeds is somewhat remarkable: he reads a history of a convulsive epidemic, and of the different opinions that have been entertained respecting it; he argues through several pages in opposition to an opinion which he insists I maintain; and after having very gravely flattered himself that he has proved the opinion which he had forced on me erroneous, after having given himself all this unnecessary trouble, he tells his readers that he is in possession of several accounts which differ materially from mine; and that I have been guilty of misrepresentation; although he does not suppose it intentional: and after having asserted this, he does not think it "proper" to put his readers in possession of any of his numerous proofs. If I have been guilty of misrepresentation, it must have been intentionally, for Mr. M'D. will recollect that I have stated in terms too plain to be misunderstood, that I was an eyewitness of what I described. Can any one imagine a person of common understanding to be guilty of such egregious folly and deception, as to publish to the world an account of a disease which had no existence but in his own fancy, (which Mr. M'D. must mean by "material difference" and "misrepresentation," if he mean any thing,) and this too when he must have been well aware that exposure to merited obloquy and contempt would have been the inevitable consequence of such an act! Such folly and deception I can only conceive to exist with a person who racks his imagination concerning the cause of a disease, of the non-existence of which he pretends to have proofs, and yet declines to avail himself of their authority.

There are two or three remarks I would wish to make on the former part of Mr. M'D.'s paper. Before, however, I proceed to these, I beg to say, I would ever carefully avoid all theological disputes.

Mr. M'Donald does not appear perfectly satisfied with the name I have given the disease "Convulsive Epidemic." How far Mr. M'D.'s correspondents have supplied the information necessary to form a correct opinion on the subject, I am not acquainted; but I presume that there is not one amongst them sufficiently hardy to deny that the individuals were convulsed. That the disorder was epidemic, or spreading, is abundantly proved by its extension over so considerable a part of this county. If he did not consider the title as adequately expressive of its nature, he should have either pointed out in what it was defective, or have proposed what he considered as a more correct denomination.

Mr. M'D. says that I have not hinted that recourse has been had in this case to medical assistance. When the disease first made its appearance, it was encouraged by a vast majority of those who witnessed it, under an impression that it was the operation of the Spirit of God upon the soul; and any attempt to interfere with respect to the affected individual, either as to assistance or advice, would have been considered as tending to prevent the conversion of a sinner; and might even have been attended with consequences dangerous to the proposer. Subsequently, however, men's minds seem more disposed to view the subject in a rational light, and some of the methodists themselves are now scrupulous in admitting it to have been produced by the cause to which they were at first inclined to ascribe it. As proofs of this, I beg to relate the following facts:

On the evening of the 2d of this month, I accidentally stopped at the door of their chapel in this town. Shortly two young women were brought out in the state in which I had had before seen many others in the same place. One was in a swoon; her respiration was slow and deep, her countenance pale and haggard, and her general appearance that of a person whose strength was exhausted. I laid my fingers on the wrist, but could scarcely distinguish the pulsation of the artery. I immediately recognised this to be the termination of a paroxysm. One of the preachers asked the bystanders whether she had been subject to these *fits?* the answer was "yes." "How long?" "Two or three months," viz. from the time the affection began to prevail in the county. After exposure to the air for some time, this patient got better. I then visited the other young lady in whom the paroxysm was (if I may judge from its violence) at its height. Several men held her on the bare ground, in the open air, and a number of females stood around her, so that I was prevented from observing her case so narrowly as I wished. I however understood that she had bitten either her tongue, or her lips, and that blood was flowing from her mouth. I heard her grinding her teeth, and saw her pull her cap off, and writhe most furiously. Some one crammed a handkerchief into her mouth to prevent her doing herself any further injury. The same preacher who stood by me when observing the former case, again took post at my elbow. He asked the same questions as before, to which like answers were given. He asked if she had been ever bled. No one could give the required information. He then observed that he had understood bleeding was sometimes advised by medical men in such cases, and when I was pointed out to him as such, he said he did not wish to obtrude an opinion, nor was he sufficiently acquainted with the nature of disease to determine the propriety of any remedy; but he only meant to say he had heard recourse was sometimes had to bleeding under similar circumstances. Now I would ask what would any one suppose was this gentleman's opinion of the affection? Is it not evident to every one, from the queries he made, that he considered these two women to labour under *disease*, and that he also imagined measures might be taken to alleviate or cure it? The following evening, talking with a sensible methodist, he candidly owned that these two cases which he himself saw greatly favoured the view I had taken of the subject.

JAMES CORNISH,

*Member of the Royal College of Surgeons.*

*Falmouth, June* 5, 1814.

